# Metabolic engineering of lactic acid bacteria for the production of industrially important compounds

**DOI:** 10.5936/csbj.201210003

**Published:** 2012-10-29

**Authors:** Maria Papagianni

**Affiliations:** a Department of Hygiene and Technology of Food of Animal Origin, Faculty of Veterinary Medicine, Aristotle University of Thessaloniki, Thessaloniki 54 124, Greece

## Abstract

Lactic acid bacteria (LAB) are receiving increased attention for use as cell factories for the production of metabolites with wide use by the food and pharmaceutical industries. The availability of efficient tools for genetic modification of LAB during the past decade permitted the application of metabolic engineering strategies at the levels of both the primary and the more complex secondary metabolism. The recent developments in the area with a focus on the production of industrially important metabolites will be discussed in this review.

## Introduction

Lactic acid bacteria are used worldwide in the industrial manufacture of fermented foods. Their most important application in this respect is in the dairy industry with an enormous variety of fermented dairy products, while next to that is the fermented meat and vegetable products industry. Besides food production, LAB are used in a variety of other industrial applications such as the production of lactic acid, high-value metabolites involved in flavor and texture development or health applications, probiotic products, and antimicrobial peptides. Characteristics such as a rather simple energy and carbon metabolism and a small genome size (∼2-3 Mb), make LAB important candidates for metabolic engineering strategies. Such strategies have mainly focused on rerouting of pyruvate metabolism to produce important fermentation end-products e.g. sweeteners, flavors, aroma compounds [1, 2], and on more complex biosynthetic pathways leading to the production of exopolysaccharides and vitamins [3, 4], while attempts to manipulate the central carbon metabolism (CCM) are rather limited in number [5]. Being one of the model organisms in microbial metabolism, *Lactococcus lactis* has been the main target of metabolic engineering among LAB. The knowledge of its complete genome sequence [6], the availability of numerous genetic tools for this microorganism [7], and its industrial relevance, facilitated its use in the development of efficient cell factories [8]. The present work aims to give an overview of the recent advances in engineering the metabolism of LAB for the production of industrially important compounds.

## 1. Lactic acid

**Figure 1:**
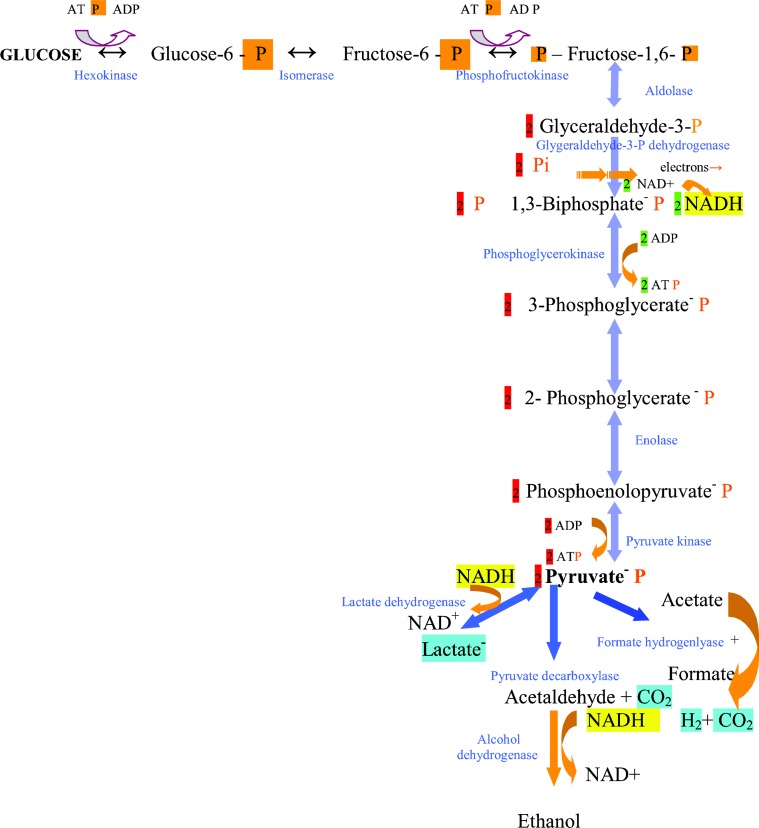
**Glycolysis (Embden-Meyerhof pathway)**. The sequence of enzymatic reactions in the conversion of glucose to pyruvate and finally, to fermentation products. In blue letters, are the enzymes involved. Highlighted, are the components exchanged between oxidation or reduction reactions. The number of the produced molecules are highlighted in red.

**Figure 2:**
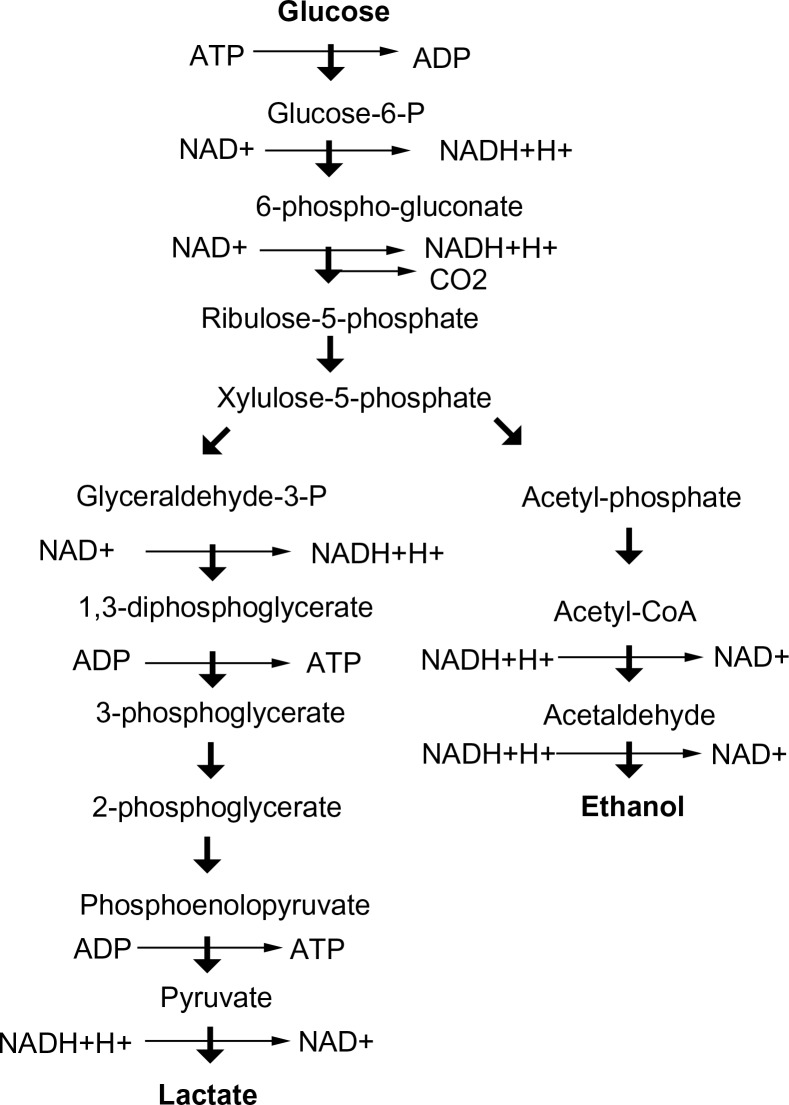
The phosphoketolase pathway.

LAB generate ATP by fermentation of carbohydrates coupled to substrate level phosphorylation. The two major pathways for the metabolism of hexoses are the glycolytic pathway (Embden-Meyerhof pathway) (Fig. 1), with lactic acid being the main end-product (homofermentative metabolism), and the phosphoketolase pathway (Fig. 2) in which other end-products such as acetic acid, propionic acid, CO_2_, ethanol and others are formed in addition to lactic acid (heterofermentative metabolism) [9]. *L. lactis* shows homofermentative metabolism in substrates with rapidly metabolized sugars with more than 90% of the metabolized sugar being converted to lactic acid. Deviation from the homofermentative mode is observed under aerobic conditions [10, 11] or during the metabolism of galactose or maltose [12, 13]. Lactic acid is used as a preservation (acidifier) and flavor-enhancing agent by the food industry, as an emulsifying and moisturizing agent by the cosmetics industry, in the synthesis of optically pure pharmaceuticals and as intermediate in pharmaceutical processes, and also by the tanning industry [14]. L-lactic acid is also used industrially as the starting material in the production of valuable synthetic biopolymers [15]. One of them, polylactic acid is expected to replace various polymers traditionally derived from the oil industry in applications ranging from fibers to packaging [16]. The rapidly growing demand for polylactic acid has led to a rapid increase in lactic acid demand worldwide [16].

An obvious target for metabolic engineering aiming at increased lactic acid production levels lies in the area of sugar utilization and the subsequent glycolytic and lactate fluxes. Regulation of glycolysis and the shift between different fermentation modes have been extensively studied with *L. lactis* [17–21]. A detailed glycolytic model for *L. lactis* based on literature information on enzyme kinetics was presented by Hoefnagel and co-workers [22]. Results published by Andersen et al. [23] for the role of phosphofructokinase (PFK) on the glycolytic flux in *L. lactis*, show that the particular enzyme plays an important role as both the glycolytic and lactate fluxes were decreased proportionally by a two-fold reduction of PFK activity. A key role of PFK with regard to the glycolytic flux control was also reported by Neves et al. [11]. Papagianni et al. [21] showed that the control of the glycolytic flux resides to a large extent in processes outside the glycolytic pathway itself, like glucose transport and the ATP consuming reactions, and allosteric properties of key enzymes like the PFK have a significant influence on the control. Following the above-mentioned work, Papagianni and Avramidis [24, 25] constructed *L. lactis* strains with altered PFK activity, by cloning the *pfkA* gene from *Aspergillus niger* or its truncated version *pfk13* that encodes a shorter PFK1 fragment, and studied the effects of increased PFK activity levels on the glycolytic capacity of *L. lactis* and lactic acid production. The results demonstrated the direct effect of PFK activity on the glycolytic flux in *L. lactis* since a two-fold increase in specific PFK activity (from 7.1 to 14.5 U/OD_600_) resulted in a proportional increase of the maximum specific rates of glucose uptake (from 0.8 to 1.7 mmol s^−1^ g CDW^−1^) and lactate formation (from 15 to 22.8 g lactate (g CDW)^−1^ h^−1^).

Lactate dehydrogenase (LDH) is the last enzyme in the pathway converting sugar to lactate in *L. lactis*. Disruption of the respective gene (*ldh*) leads to diversion of the majority of pyruvate towards mixed-acid products [26, 27]. Andersen and co-workers [28] studied the extent of the control of LDH on metabolic fluxes in wilde-type *L. lactis* cells through construction of a series of mutant strains with LDH activities ranging from below 1% to 133% of the wild-type activity level. Flux control coefficients were estimated to reveal that LDH did not exert any control on the glycolytic flux at the wild-type enzyme level and also not on the flux catalyzed by the enzyme itself, i.e. on lactate production.

Lactic acid can be produced by LAB in its L- or D-isomer form. L-lactic acid is preferred for food and pharmaceutical applications and as starting material in the production of biopolymers, while D-lactic acid is toxic for humans. Therefore, metabolic engineering studies have focused on the production of pure L-lactic acid by homofermentative LAB. Inactivation of the D-lactate dehydrogenase gene (*ldhD*) by chromosomal integration in the work of Bhowmik and Steele [29] resulted in the formation of pure L-lactic acid in *Lactobacillus helveticus*, although in amounts comparable to that obtained by the wild type strain. Working also with *Lb. helveticus*, Kylä-Nikkilä and co-workers [30] constructed two stable *ldhD*-negative strains: one carrying an additional copy of the *ldhL* gene under the control of the *ldhD* promoter and the other with deleted the *ldhD* promoter region. Improvement of L-lactic acid production by almost 20% was achieved in the first case under low pH conditions. Similarly, inactivation of *ldhD* in *Lb. johnsonii* resulted in the production of pure L-lactate [31]. As with *Lb. helveticus*, overexpression of the *ldhL* gene in *Lb. plantarum* had only a small effect on the production of L-lactate [32]. Davidson et al. [33] working with *L. lactis* increased the copy number of the *las* operon genes, which apart from *pfk* includes pyruvate kinase (*pyk*) and the *ldhL* genes, and reported only a small increase in L-lactic acid production. The construction of an L-lactate overproducing *L. lactis* strain by UV mutagenization was reported by Bai and co-workers [15]. Overproduction of L-lactate in that case was through a reduction of NADH oxidase activity and increase of the glucose uptake rate.

## 2. Flavor formation

### 2.1. Diacetyl

Diacetyl is naturally produced by LAB, especially *L. lactis* biovar. *diacetylactis*, from citrate in co-fermentation with lactose. The intermediate product of the fermentation α-acetolactate, is the chemical precursor for diacetyl to which can be converted through an oxidative decarboxylation reaction. Citrate-utilizing LAB produce diacetyl during milk fermentation in the production of butter, buttermilk and several cheeses while diacetyl generates the typical butter aroma in these products [34]. Because of its value as aroma compound, efficient production of diacetyl from lactose rather than citrate has been the aim of several metabolic engineering strategies.

Platteeuw et al. [26] constructed an *ldh*-deficient strain which under aerobic conditions showed a remarkable shift in pyruvate metabolism leading to increased acetate and acetoin production. α-acetolactate is formed as an unstable intermediate molecule during this process. In another attempt, *ldh* inactivation was combined with overexpression of the α-acetolactate synthase gene (*als*) that encodes α-acetolactate synthase (ALS) [35]. The result of the engineering was the production of very high amounts of acetoin and rather low amounts of α-acetolactate. ALS is one of the two enzymes that catalyze the conversion of pyruvate to α-acetolactate, the other being the acetohydroxy acid synthase (ILVBN, encoded by the *ilvBN* gene). ILVBN is an anabolic synthase involved in branced chain amino acid synthesis, while ALS acts as a catabolic synthase. Overexpression of the *ilvBN* gene in *L. lactis* grown under aerobic conditions resulted in a 4-fold increased flux towards acetoin [35]. Another strategy involved cloning the *als* gene on a multi-copy plasmid and resulted in 100-fold increase in ALS levels and a 40% rerouting efficiency of the pyruvate pool towards acetoin under aerobic conditions [26]. The above described approaches have been rather unsuccessful in constructing strains that produce efficiently diacetyl. The activity of α-acetolactate decarboxylase gene (*αldB*) that encodes the ALDB enzyme which catalyzes the conversion of α-acetolactate to acetoin, was still present in those strains. However, attempts with inactivated *αldB* gene resulted in low diacetyl levels under aerobic conditions which were increased by overexpression of the *ilvBN* genes [36].

Lopez de Felipe et al. [37] reported a novel approach for metabolic engineering *L. lactis* by controlled expression of the NADH oxidase (NOX) (co-factor engineering). NADH oxidase-overproducing strains were constructed using the nisin inducible expression (NICE) system [37].Overproduction of NOX at regulated aeration levels permitted the effective control of the NADH:NAD^+^ ratio and resulted in the complete shift of the fermentation pattern from homolactic to mixed acid. Introduction of this system to a strain with inactivated *αldB* gene [39] led to the efficient conversion of the native pyruvate metabolism towards α-acetolactate and diacetyl. The combination of NOX overproduction with ALDB inactivation under aerobic conditions proved to be a very successful strategy for diacetyl production through metabolic engineering. Within the same area of co-factor engineering, most recently, Guo and co-workers [40] reported a novel strategy to precisely control the pyruvate distribution for fine tuning of lactate and diacetyl production in *L. lactis* through promoter engineering. Using selected promoters for the constitutive expression of the NADH oxidase gene, NOX activity increased to 58.17-fold of the wild-type strain under aerobic conditions. Via an altered NADH/NAD^+^ ratio, the reduced pyruvate to lactate flux was rerouted to diacetyl and production increased from 1.07±0.03 mM to 4.16±0.06 mM.

Hoefnagel and co-workers [41] adopted a more rational combination strategy consisting of a detailed kinetic model of the branches around pyruvate metabolism in *L. lactis* and metabolic control analysis. The presented model (available at http://jjj.biochem.sun.ac.za) showed that the enzymes with the greatest effect on the flux to diacetyl reside outside the ALS branch itself. Model predictions were confirmed by experiments, i.e. deletion of *ldh* and overexpression of NOX increased the flux through the ALS branch from 0 to 75% of measured product formation rates.

Improved diacetyl producing *L. lactis* strains were designed by Oliveira et al. [42] through enhanced metabolic engineering strategies. These strategies involved genome-scale flux models able to simulate and analyze network capabilities and cell function in continuous cultures performed under aerobic or anaerobic conditions. As mathematical frameworks for modeling were used flux balance analysis (FBA) and minimization of metabolic adjustment (MOMA).

### 2.2. Acetaldehyde

Acetaldehyde, an important aroma compound in dairy products, can be produced by LAB through several pathways. An important metabolic precursor for acetaldehyde synthesis by LAB is glucose through the pyruvate and acetyl-CoA intermediates of glycolysis [43]. Amino acids and other metabolites that are converted to pyruvate may also contribute to acetaldehyde biosynthesis. The most important pathway however is through the conversion of threonine into acetaldehyde and glycine, a reaction catalyzed by threonine aldolase [44, 45]. In *Streptococcus thermophillus* the enzyme with threonine aldolase activity is serine hydroxymethyltransferase (SHMT) encoded by the *glyA* gene. Overexpression of *glyA* in *S. thermophillus* strains by Chaves et al. [45] resulted in overproduction of acetaldehyde by 80-90% compared to parental strains. Another attempt to improve acetaldehyde production through metabolic engineering was reported by Bongers et al. [46] for *L. lactis*. In that work, overexpression of pyruvate decarboxylase (*pdc*) from *Zygomonas mobilis* in *L. lactis* rerouted pyruvate metabolism towards acetaldehyde. The overexpressed gene controlled by the NICE system, competes with lactate dehydrogenase (*ldh*) for the available pyruvate. To further increase pyruvate availability, the NADH oxidase gene (*nox*) was also overexpressed. The double mutant converted almost 50% of the consumed glucose to acetaldehyde under anaerobic conditions.

## 3. Production of sweeteners

### 3.1. L-alanine

L-alanine is used as a food sweetener and in pharmaceutical applications. Its efficient production by *L. lactis* is an example of the successful introduction of a heterologous pathway in this organism for the production of interesting new products. Conversion of pyruvate into alanine takes place in various anaerobic bacteria and involves a single enzymatic step catalyzed by alanine dehydrogenase. The pathway was introduced in *L. lactis* through cloning and expression of the *alaD* gene of *Bacillus sphaericus* that encodes for L-alanine dehydrogenase (ALAD) - with kinetic properties similar to those of LDH of *L. lactis* [27]. *alaD* was cloned under the control of the *nisA* promoter in *L. lactis*. Its overexpression resulted in a 30-40% rerouting efficiency from lactate toward alanine when resting cells grown on glucose were used in the presence of excess of ammonium which is required for the conversion of pyruvate to alanine by ALAD. Furthermore, introduction of the system into LDH-deficient lactococcal cells resulted in the complete conversion of glucose into alanine (>99%) by changing the metabolism of *L. lactis* from homolactic to homoalanine [27]. Complete conversion of glucose into L-alanine by the LDH-deficient *L. lactis* was achieved by inactivation of the alanine racemace gene (*alr*). This example illustrates how the homofermentative metabolism of *L. lactis* can be efficiently redirected into the production of a commercially important compound.

### 3.2. Mannitol

Mannitol, a six-carbon sugar alcohol, is produced commercially by catalytic hydrogenation from glucose/fructose mixtures and is used in the food and pharmaceutical industries, as well as in medicine. The chemical process yields only a 25-30% of mannitol from the total sugars used, as the main product of the process is sorbitol. Separation of mannitol and sorbitol is relatively difficult and the requirement for separation results in increased production costs and lower yields [47]. Microbially, mannitol is synthesized by many eukaryotes (fungi and yeasts) and a few bacteria, mainly heterofermentative LAB, without the co-formation of sorbitol (homofermentative LAB produce also mannitol but in very low levels) [47]. These LAB are known to convert fructose to mannitol by using mannitol dehydrogenase (MDH). In this reaction, part of the fructose is directed to the heterofermentative pathway while the rest acts as an electron acceptor and is reduced to mannitol [48]. Mannitol production is increased in this reaction if fructose is co-fermented with glucose [48]. Increased mannitol yields have been achieved by optimizing the mannitol fermentation of heterofermentative LAB [49].

Heterofermentative LAB may produce mannitol from fructose in excellent quantities (up to 66% have been reported) however, this is always co-produced with lactic and acetic acids and small quantities of other metabolites. Therefore, development of strains that overproduce mannitol, or produce co-products in limited amounts or even do not produce them, would be desirable and research interest has directed towards metabolic engineering approaches for mannitol production. The so far reported attempts are limited to those by Aarnikunnas et al. [50] and Helanto et al. [51]. In the first case, through inactivation first of the *ldhD* and then of the *ldhL* gene, a strain of *Lb. fermentum* was constructed that was able to produce mannitol and either L-lactate or mannitol and pyruvate in a single process. The process was anaerobic and the recombinants produced 2,3-butanediol as co-product. In the second case of Helanto et al. [51], the yield of mannitol from fructose from *Leuconostoc pseudomesenteroides* was improved from 74 to 85% by applying random mutagenesis to the producer organism for inactivating its fructokinase activity.

Several strategies have also been reported for enhancing mannitol production from homofermentative LAB, e.g. *L. lactis* and *Lb. plantarum*. Neves et al. [52] reported the synthesis of mannitol and mannitol-1-phosphate (Mtl1P) in *ldh*-deficient *L. lactis* strains. Production of mannitol was rationalized as an alternative way to satisfy the redox balance during the catabolism of glucose since the conversion of fructose-6-phosphate (F6P) to Mtl1P is associated with NAD^+^ regeneration. It was also observed in that work that the produced mannitol was rapidly taken up and metabolized after the depletion of glucose and therefore it was clear that a metabolic strategy for increased mannitol production should involve inactivation of the mannitol transport system. This was achieved in a later work by the same team [53]. The mannitol transport system was inactivated in *ldh*-deficient *L. lactis* by deleting the *mtlA* or *mtlF* genes encoding EIICB^Mtl^ and EIIA^Mtl^, respectively. In the resting state, the double mutant strains converted almost 30% of glucose to mannitol. Wisselink et al. [54] achieved the highest reported conversion yield from glucose to mannitol (50%) with *ldh*-deficient *L. lactis* in which the mannitol 1-phosphate dehydrogenase gene (*mtlD*) of *Lb. plantarum* and the mannitol 1-phosphate phosphatase gene of the protozoan parasite *Eimeria tenella* were overexpressed. The 50% yield obtained by growing cells is close to the theoretical yield of mannitol of 67% in *L. lactis*.

### 3.3. Sorbitol

Sorbitol, like mannitol, is a six-carbon sugar alcohol with applications in the food and pharmaceutical industries. It is traditionally produced by catalytic hydrogenation of glucose [51] while only a few organisms have been described as able to produce it. Early studies of biotechnological production of sorbitol focused on the Gram^-^ bacterium *Zygomonas mobilis* which can convert fructose and glucose to sorbitol for osmoprotection [55].

In LAB, sorbitol production through metabolic engineering has been reported with *Lb. plantarum* and *Lb. casei*. Mannitol phosphate dehydrogenase (MPDH) and LDH were inactivated in a *Lb. plantarum* strain overexpressing a sorbitol dehydrogenase (SDH) gene (*stlDH*). As both MPDH and SDH use fructose-6-phosphate (F6P) as substrate, the inactivation of MPDH allowed more F6P to be reduced to sorbitol-6-P by SDH. The result, in resting cells with glucose as substrate, was a near to theoretical yield of sorbitol: 0.65 mol/mol glucose [56]. Nissen et al. [57] constructed a *Lb. casei* strain in which the sorbitol-6-P-dehydrogenase gene (*gutF*) was integrated into the chromosomal lactose (*lac*) operon. The gene was under the control of the *lac* operon which is repressed by glucose and induced by lactose. Resting recombinant cells pre-grown on lactose produced 0.024 mol sorbitol/mol glucose while the parental strain produced negligible amounts. In addition, deletion of *ldhL* further increased sorbitol production to 0.043 mol sorbitol/mol glucose, presumably because of elevated NADH pools in the cells which increased the conversion rate of glucose to sorbitol.

### 3.4. Xylitol

Xylitol is a five-carbon sugar alcohol currently produced through the chemical reduction of xylose [58]. Since yeasts, fungi and bacteria are able to reduce D-xylose to xylitol by xylose reductase, their potential for microbial production of xylitol has been investigated and in many cases, especially with yeasts, metabolic engineering efforts have been undertaken [58].

LAB are not reported to produce xylitol naturally although strains of *S. avium* and *Lb. casei* are able to metabolize it [59]. Nyyssölä et al. [60] constructed a recombinant *L. lactis* strain in which the xylose reductase (XR) gene from *Pichia stipitis* and a xylose transporter from *Lb. brevis* were expressed. This co-expression however did not improve xylitol production. In spite of this, an increased productivity level, comparable to that of the more efficient yeast producers, was achieved in fed-batch fermentation by using non-growing *L. lactis* cells.

## 4. Production of exopolysaccharides (EPS)

Production of EPS by lactic acid bacteria with wide use by the dairy industry such as *Lactococcus* and *Streptococcus*, is an attractive subject of metabolic engineering [61]. In situ EPS synthesis allows modulation of rheology and improved sensory properties e.g. mouthfeel and texture of food products, as well as acquisition of some health promoting properties (prebiotics). Natural production of EPS by LAB is very low compared to the production of other food-grade EPS (e.g. xanthan, gurdlan) produced by non-dairy bacteria. Metabolic engineering strategies for improvement of EPS production by LAB have mainly focused on precursor synthesis. Sugar nucleotides, such as UDP-glucose, UDP-galactose and TDP-rhamnose, are synthesized from glucose-1-phosphate (G1P) as a general precursor. The conversion of the intermediate G6P to G1P by phosphoglucomutase (PGM) and the synthesis of UDP-glucose from G1P catalyzed by UDP-glucose phosphorylase (GalU) are control points in EPS production. Overexpression of GalU under the control of a nisin inducible promoter increased the specific activity of the enzyme by 20-fold in *L. lactis* which in turn increased both UDP-glucose and UDP-galactose synthesis by 8-fold although EPS synthesis was not significantly enhanced [62]. However, overexpression of PGM and GalU in *S. thermophillus* led to a 2-fold increase in EPS synthesis [63].

A different approach aims at structural engineering of EPS produced by LAB. This can be achieved either by controlling the culture conditions (e.g. the type of sugar source) or by genetic engineering strategies [64]. The potential for controlling the formation of EPS structure by introducing new or existing glycosyltransferases into LAB has been reported by Boels et al. [65].

## 5. Production of vitamins

Increased production of the B-vitamins folate and riboflavin (B2) by LAB has been another target area of metabolic engineering. These vitamins are essential co-factors in important metabolic activities of the producer cell (and all living cells) such as the biosynthesis of amino acids and nucleic acids and they are produced by several LAB and propionic acid bacteria often in large quantities and enrich fermented foods. It has been shown that metabolic engineering can be used to increase folate levels in *L. lactis* [66, 67], *Lb. gasseri* [68] and *Lb. reuteri* [69]. Several of folate biosynthetic genes have been overexpressed individually or in combination in *L. lactis* strain NZ9000 using the NICE system [70]. Overproduction of the first enzyme in folate biosynthesis, GTP cyclohydrolase, led to a 3-fold increased production of folate. Overexpression of *folKE* that encodes the biprotein 2-amino-4-hydroxy-6-hydroxymethyldihydropteridine pyrophosphokinase and GTP cyclohydrolase I in *L. lactis* subsp. *cremoris* MG1363, was found to increase the extracellular folate production almost 10-fold and the total production 3-fold [66]. Increases in folate production can be also achieved by overexpressing other genes involved in the biosynthetic pathway of related metabolites. For example, the overproduction of para-aminobenzoic acid (*p*ABA) did not lead to elevated folate pools on its own. However, simultaneous overexpression of the *p*ABA and folate biosynthesis gene clusters reached high folate levels which did not depend on *p*ABA supplementation [67]. The overproduction of *p*ABA led to relatively low intracellular folate pools and a relatively high secretion of folate.

The riboflavin biosynthetic (*rib*) operon was identified in *L. lactis* subsp. *cremoris* strain NZ9000 [71]. The strain was converted from a riboflavin consumer into a rivoflavin ‘factory’ by overexpressing its riboflavin biosynthetic genes [71]. Substantial riboflavin overproduction was reported when all four biosynthetic genes (*ribG*, *ribH*, *ribB* and *ribA*) were overexpressed simultaneously in *L. lactis*.

By directed mutagenesis followed by selection and metabolic engineering, Sybesma and co-workers [4] modified the biosynthetic pathways of folate and riboflavin in *L. lactis* resulting in the simultaneous overproduction of both vitamins. According to the authors, novel foods, enriched through fermentation using these multivitamin-producing starters, could compensate the B-vitamin-deficiencies that are common even in highly developed countries.

## Conclusions

The cases presented shortly in this review reveal a variety of approaches for metabolic engineering of LAB including mutagenesis, classical gene inactivation and overexpression, redox engineering or engineering of primary carbon metabolism, as well as predictive approaches for improving cellular phenotypes. They also exemplify the power of metabolic engineering in LAB for the improved and often efficient production of a number of industrially important metabolites with wide applications in the food and pharmaceutical industries. It is expected however that progress in metabolic modelling and Systems Biology approaches will provide the means for engineering complex (biosynthetic) pathways for the efficient production of metabolites such as vitamins, antioxidants and other nutraceuticals by LAB.
